# Sustained Elevation of Intraocular Pressure Associated with Intravitreal Administration of Anti-vascular Endothelial Growth Factor: A Systematic Review and Meta-Analysis

**DOI:** 10.1038/srep39301

**Published:** 2016-12-21

**Authors:** Yandan Zhou, Minwen Zhou, Shigang Xia, Qiancheng Jing, Ling Gao

**Affiliations:** 1 Department of Ophthalmology, the Second Xiangya Hospital, Central South University, Changsha, Hunan 410011, China; 2 Department of Ophthalmology, Shanghai First People’s Hospital, School of Medicine, Shanghai Jiao Tong University, Shanghai, China; 3 Department of Ophthalmology, the second hospital affiliated to the University of South China, Hengyang, Hunan, China; 4 Department of Otorhinolaryngology, Xiangya Hospital,Central South University, Changsha, Hunan, China

## Abstract

This study aimed to assess whether repetitive intravitreal injections (IVI) of anti-vascular endothelial growth factor (anti-VEGF) cause sustained elevation of intraocular pressure (SE-IOP). We conducted a systematic review and meta-analysis based on five randomized controlled trials (RCTs) assessing 1428 subjects and 17 non-RCTs evaluating 8358 cases. In the RCTs, an increased risk of SE-IOP was found in the anti-VEGF group (summary risk ratio [RR] = 3.00, 95% confidence interval [CI]: 1.63–5.53) compared with the sham injection or laser group. The increased risk of SE-IOP was correlated with follow-up duration (RR = 2.14, 95% CI 0.69–6.57 at 6 months; RR = 3.15, 95% CI 0.99–10.09 at 12 months; RR = 3.48, 95% CI 1.38–8.78 at 23 months). The risk of SE-IOP after non-exclusion of pre-existing glaucoma patients (RR = 3.48, 95% CI 1.38–8.78) was higher than that obtained after excluding pre-existing glaucoma patients (RR = 2.6, 95% CI 1.16–5.81). In non-RCTs, the pooled prevalence of SE-IOP was 4.7% (95% CI 3.7–5.8) regardless of diagnosis criteria. In conclusion, repeated intravitreal injections of anti-VEGF agents cause a 2-fold elevation in SE-IOP risk.

Currently, intravitreal injection of anti-VEGF is typically applied in the treatment of choroidal neovascularization (CNV), which occurs in patients with wet age-related macular degeneration (wAMD) and high myopia. It is also used to treat patients with macular edema secondary to diabetic retinopathy (DME) and retinal vein occlusions (RVO-ME). Ranibizumab (a recombinant, humanized monoclonal antibody targeting VEGF-A), Bevacizumab (a full-size humanized monoclonal antibody targeting VEGF-A), and aflibercept (a soluble decoy receptor fusion protein)^1^,^2^,^3^, are commonly used for the treatment of CNV and macular edema. Pegaptanib, a RNA aptamer targeting VEGF165, is currently used for the treatment of AMD patients^4^,^5^, but has not yet been approved for macular edema secondary to RVO^6^.

IOP usually increases immediately after anti-VEGF intravitreal injection, before returning to baseline within 30 to 60 minutes^7^,^8^,^9^,^10^,^11^. The transient elevation of IOP is mainly related to acute volume expansion of the eyeball, which can be prevented by prophylactic anterior chamber paracentesis^12^,^13^. However, there is controversy regarding long-term SE-IOP. Multiple studies have reported SE-IOP is related with the intravitreal injection of anti-VEGF agents^14^,^15^,^16^, while others hold different views^17^,^18^. To the best of our knowledge, no systematic review or meta-analysis highlighting the association of repeated intravitreal injections of anti-VEGF with SE-IOP is available. Therefore, we performed a meta-analysis to assess the risk of SE-IOP, exploring its possible impacts on patient outcomes.

## Results

### Article Characteristics and Study Categorization

**Figure 1:**
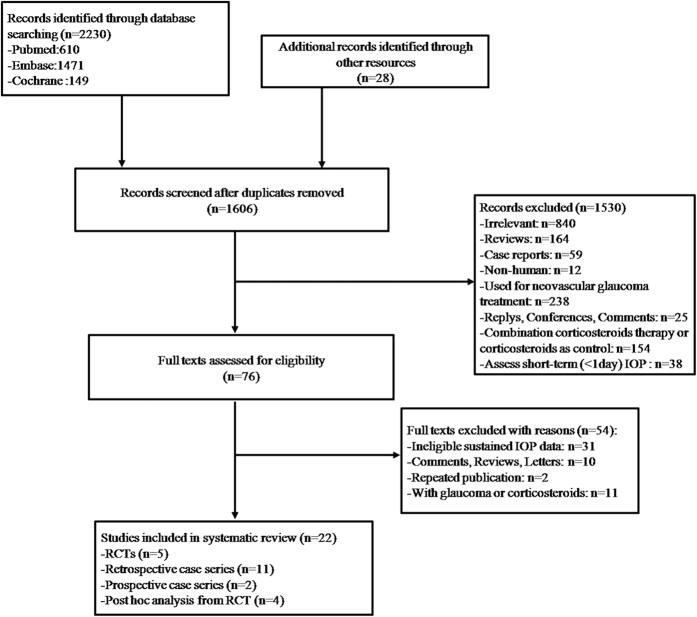
Flow diagram of the assessment of studies identified in this systematic review and meta-analysis.

The literature search yielded 2258 articles, including 610, 1471, 149, and 28 from PubMed, EMBASE, the Cochrane Library, and a manual search, respectively. Because there are no uniform criteria for SE-IOP, two cases can be considered SE-IOP: IOP ≥21 mmHg or 5 mmHg higher than the baseline for at least three weeks on two visits, and IOP >25 mmHg on a single visit with anti-glaucoma therapy requirement. Twenty-two studies were included after removal of 652 duplicated reports and 1584 ineligible articles (Fig. 1). There were five RCTs^19^,^20^,^21^,^22^,^23^ and 17 non-RCT studies^14^,^15^,^16^,^17^,^24^,^25^,^26^,^27^,^28^,^29^,^30^,^31^,^32^,^33^,^34^,^35^,^36^. The latter group included 11 retrospective case series^14^,^17^,^24^,^25^,^26^,^27^,^28^,^29^,^30^,^31^,^32^, two prospective studies^33^,^34^, and four post hoc analyses^15^,^16^,^35^,^36^. Follow-up in the included articles ranged from 2.26^27^ to 60^31^ months. Detailed characteristics of comparative and non-comparative studies are provided in Table 1 and Table 2, respectively.

### Association of SE-IOP with Intravitreal Injection of Anti-VEGF in RCTs

**Figure 2:**
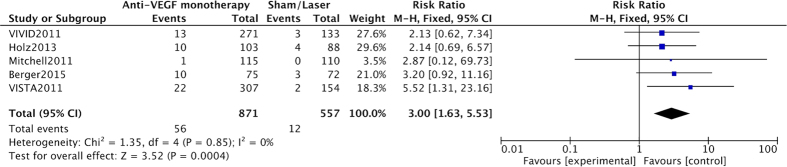
Forest plot showing the association of intravitreal anti-VEGF mono-therapy with risk of sustained elevation of IOP (SE-IOP) with a fixed-effects model. M-H, Mantel Haenszel statistics; RR, risk ratio; CI, confidence interval.

All the RCTs^19^,^20^,^21^,^22^,^23^ were included comparing anti-VEGF mono-therapy and control (sham injection or laser treatment) groups. Follow-up of the included cases in all RCTs was at least three months. Anti-VEGF significantly increased the risk of SE-IOP (summary RR = 3.00, CI 1.63–5.53, P = 0.0004; Fig. 2). No significant heterogeneity was found among the above studies (*I*^2^ = 0%), and no study significantly influenced the overall effect in sensitivity analysis.

**Figure 3:**
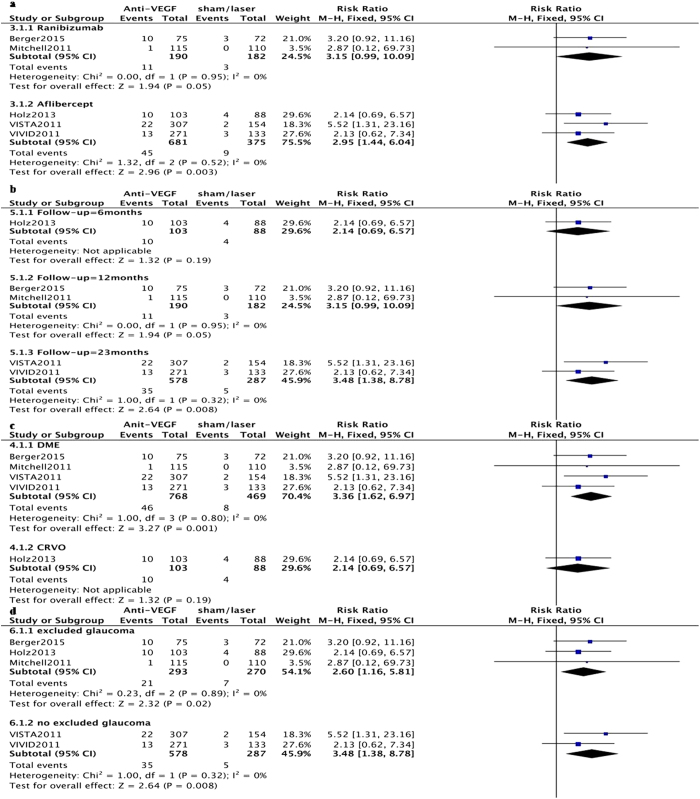
Forest plot showing the association of intravitreal anti-VEGF mono-therapy with risk of sustained elevation of IOP (SE-IOP) in various subgroups with a fixed-effects model. (**a**) Different agents: RRs for ranibizumab and aflibercept; (**b**) Different follow-up periods: RRs at 6, 12, and 23 months; (**c**) Different diseases: RRs for diabetic macular edema (DME) and central retinal vein occlusion (CRVO); (**d**) Exclusion of pre-existing glaucoma: RRs for exclusion and non-exclusion of pre-existing glaucoma patients. M-H, Mantel Haenszel statistics; RR, risk ratio; CI, confidence interval.

Subgroup-analyses of all RCTs for anti-VEGF types (Fig. 3a), different follow-up periods (Fig. 3b), ocular diseases (Fig. 3c), and exclusion/inclusion of patients with pre-existing glaucoma (Fig. 3d) were performed. Interestingly, the risk of SE-IOP increased with follow-up duration, with RRs at 6, 12, and 23 months of 2.14 (CI 0.69–6.57, P = 0.19), 3.15 (CI 0.99–10.09, P = 0.05), and 3.48 (CI 1.38–8.78, P = 0.008), respectively. Meanwhile, patients with pre-existing glaucoma were more vulnerable to anti-VEGF; indeed, a RR of 2.6 (CI 1.16–5.81, P = 0.02) was obtained for studies excluding pre-existing glaucoma, while 3.48 (CI 1.38–8.78, P = 0.008) was found in those including patients with pre-existing glaucoma.

### SE-IOP Prevalence is Independent of Diagnostic Criteria in Non-RCTs

**Figure 4:**
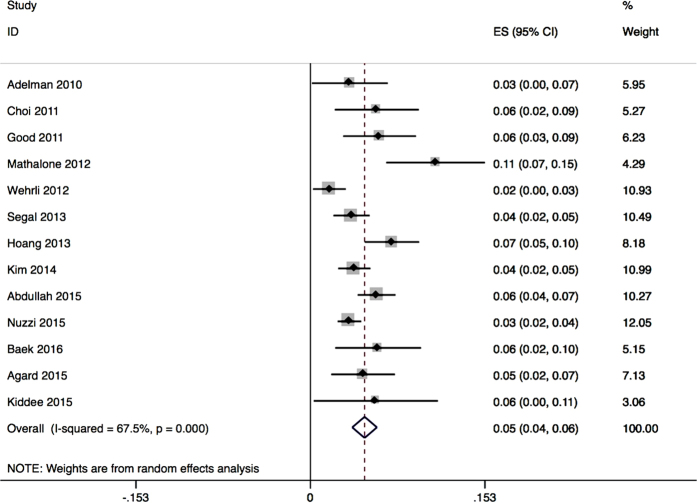
Pooled prevalence of SE-IOP assessed with a random effects model.

Eleven retrospective^14^,^17^,^24^,^25^,^26^,^27^,^28^,^29^,^30^,^31^,^32^ and two prospective^33^,^34^ studies, which reported the case numbers and incidence rates of SE-IOP among the 5062 subjects, were analyzed. Prevalence of SE-IOP varied from 1.6% (5 of 302 cases) to 11% (22 of 201 case), with a pooled prevalence (Fig. 4) of 4.7% (238/5,062 cases; (CI 3.7–5.8) in a random effects model, with significant heterogeneity between the studies (*I*^2^ = 67.5%). Egger’s test (P = 0.726) indicated no evidence of publication bias (Fig. S1). Among the 2366 subjects who received intravitreal injection of bevacizumab in eleven studies^14^,^17^,^24^,^25^,^26^,^28^,^29^,^30^,^31^,^32^,^33^, 104 developed SE-IOP, with a pooled prevalence of 3.8% (CI 2.3–5.4) in a random effects model, with significant heterogeneity (*I*^2^ = 70.1%) (Fig. S2). Sixty-nine of 1649 subjects^17^,^24^,^25^,^26^,^29^,^30^,^31^,^33^ suffered from SE-IOP after intravitreal injection of ranibizumab, with a pooled prevalence of 3.5% (CI 2.2–4.8) in a random effects model, with moderate heterogeneity (*I*^2^ = 44.4%) (Fig. S3).

### Stratified Analyses and Sensitivity Analysis of SE-IOP Prevalence in Post hoc Analyses

Four post hoc analyses were carried out in the included RCTs. Bakri *et al*.^15^ analyzed the prevalence of SE-IOP by comparing intravitreal Ranibizumab treatment versus control. Prevalence rates were39.9% versus 29.1% with IOP ≥21 mmHg, 10.9% versus 5.1% with IOP ≥25 mmHg, and 26.1% versus 13.6% with IOP ≥21 mmHg and ≥6 mmHg above baseline. Boyer *et al*.^16^ similarly compared intravitreal pegaptanib with sham treatment and reported prevalence rates of 24.6% versus 21.5% with ≥22 mmHg on 1 visit, 7.0% versus 7.5% with ≥24 mmHg on 1 visit, but 5.3% versus 9.3% with ≥22 mmHg on 2 visits. Freund *et al*.^36^ reported 7.9% as a comparable prevalence for intravitreal ranibizumab with IOP >21 mmHg, 5.9% with IOP ≥25 mmHg, 18.6% with IOP ≥5 mmHg above baseline, and 1.3% with IOP ≥10 above baseline mmHg. Bressler *et al*.^35^ reported the cumulative probability of SE-IOP (defined as IOP ≥22 mmHg with an increase of ≥6 mmHg from baseline on 2 consecutive visits, or initiation or augmentation of ocular hypotensive therapy, through 3 years of follow-up) to be 9.5% after combined treatment with ranibizumab and laser group, while 3.4% was found in the laser group.

A limitation of this analysis of SE-IOP prevalence was the significant heterogeneity of non-RCTs, as shown in Fig. 4. Therefore, stratified analyses were performed for different disease subgroups, sample sizes, diagnostic criteria and sustained times of SE-IOP (Table 3). No heterogeneity were found within the two former subgroups. However, low heterogeneity was obtained when using different criteria to define sustained elevated IOP. The incidence of SE-IOP also varied with different criteria. Indeed, SE-IOP incidence was highest for the criterion of IOP ≥22 mmHg and ≥6 mmHg above baseline, with a value of 8.3% (CI 3.5–13.1) and high heterogeneity (*I*^2^ = 68.8%, P = 0.073). SE-IOP incidence was highest in the 4–6 week sustained time subgroup, with a value of 5.8% (CI 4.4–7.1) and high heterogeneity (*I*^2^ = 52.8%, P = 0.038).

## Discussion

To the best of our knowledge, this is the first systematic review and meta-analysis assessing the risk of sustained IOP elevation following intravitreal injection of anti-VEGF. Meta-analysis of five RCTs revealed that repeated intravitreal injection of anti-VEGF agents increases the risk of SE-IOP compared to controls. Even when taking into consideration the impacts of drug type, disease conditions, length of the follow-up period, and exclusion of pre-existing glaucoma and corticosteroids, the outcomes were consistent. SE-IOP prevalence was 4.7% overall, with a narrow confidence interval that varied from 2 to 11% regardless of diagnosis criteria. Compared to the well-known anti-VEGF study by MARINA and ANCHOR^15^, which reported a prevalence of 26.1% for SE-IOP, this study found a pooled prevalence of 8.3% with criteria of IOP above 22 mmHg and 6 mmHg above baseline on 2 visits during follow-up. Two studies^17^,^30^ described the time interval from the intravitreal injection to development of peak SE-IOP, with median values of 14 weeks in Wehrli *et al*. and 7 months in Abdullah *et al*. The assessed agents were ranibizumab, bevacizumab or both. According to the studies which reported SE-IOP outcomes, the majority of patients with SE-IOP need anti-glaucoma drugs to lower IOP, with few requiring anti-glaucoma surgeries^14^,^25^.

The mechanisms of SE-IOP by intravitreally injected anti-VEGF remain unknown. They may involve 1) Intraocular injection-related complications, such as mechanical blockage of the trabecular meshwork or Schlemm’s canal outflow pathways^37^,^38^ caused by anti-VEGF agents, byproducts of pharmacologic compounding or storage, or chronic changes from recurrent episodes of transient post-injection elevations in IOP^39^. 2) Drug-induced complications such as trabeculitis or uveitis^40^ and cytotoxicity to the trabecular meshwork in the presence of bevacizumab at high concentrations, as demonstrated *in vitro*^41^. In the current study, pooled prevalence of SE-IOP was 3.8% (CI 2.3–5.4) in bevacizumab and 3.5% (CI 2.2–4.8) in ranibizumab. Bressler and Bakri^15^,^35^ believed that the risk of SE-IOP after repeated IVR is relatively low, although ocular hypotensive treatment may be needed in some patients. Studies evaluating the prevalence of SE-IOP caused by aflibercept are scarce. Freund^36^ reported that elevated IOP incidence is lower in the IVI Aflibercept group than in patients treated IVI with Ranibizumab. Prevalence rates of SE-IOP caused by Bevacizumab and Ranibizumab were 9.9% (10/101) versus 3.1% (3/96) in Good’ study, and 4.76% (32/672) versus 0 (0/4) in Abdullah’ report^26^,^30^. We did not statistically compared prevalence rates of SE-IOP caused by bevacizumab and ranibizumab due to the limitation of non-RCT data and large study heterogeneity. 3) Patients with pre-existing glaucoma or not. Even when controlling IOP before injection, individuals with or without pre-existing glaucoma showed different responses to intravitreally injected anti-VEGF in this meta-analysis. RCT forest plots yielded RRs of 2.6 (CI 1.16–5.81) with excluded pre-existing glaucoma, and 3.48 (CI 1.38–8.78) in the non-excluded subgroup, suggesting the need for IOP monitoring, especially in glaucoma patients. Five non-RCTs supported this observation. In cases without pre-existing glaucoma versus those with pre-existing glaucoma, incidence rates of sustained IOP elevation were 11.3% (21 in 186) versus 6% (1 in 15) in Mathalone *et al*.^14^, 1.1% (3 in 270) versus 6.2% (2 in 32) in Wehrli *et al*.^17^, 3.1% (6 in 194) versus 33% (7 in 21) in Good *et al*.^26^ and 3.2% (6 in 186) versus 12.9% (4 in 31) in Agard *et al*.^33^, respectively. The reverse trend in Mathalone’s study may be related to the limitation of small sample size for pre-existing glaucoma cases (1 in 15). In Kim’s study^29^, among the 27 treated eyes with IOP elevation, 8 eyes had glaucoma at baseline. We speculated that SE-IOP may have some association with pre-existing glaucoma types. In neovascular glaucoma (NVG) cases, SE-IOP may be the natural course of NVG despite the inhibitory effects of anti-VEGF on intraocular neovascularization to some extent. In non-NVG cases, it is possible that eyes with an already compromised aqueous humor outflow system are more prone to developing elevated IOP^26^,^29^.

The dose-response relationship between the number of IVIs and risk of SE-IOP induced by anti-VEGF was amphibolous. Three studies^17^,^29^,^42^ supported this relationship, but we could not pool the data for different effect sizes, and one of them lacked a detailed case number. Hoang^42^ reported the hazard ratio (HR) of the 29^th^ bevacizumab and/or ranibizumab injection is much higher than that of the 12^th^ injection. For unilaterally injected patients receiving ≤12 injections, the frequency of SE-IOP in the treated eyes was found to be very close to that of untreated control eyes. In the subset of unilaterally treated eyes which received ≥29 injections, the frequency of SE-IOP in treated eyes (8.7%, 4/46) was much higher than that of untreated eyes (0%, 0/46). It was identified that a greater number of intravitreal anti-VEGF injections is associated with an increased risk of sustained IOP elevation. However, Kim^29^ reported SE-IOP frequencies of 4.3%, 2.9%, 4.7% and 2.6% in patients receiving 3–5 total injections, 6–8 total injections, 9–14 total injections and 15–37 total injections (bevacizumab and/or ranibizumab), respectively, with no statistically significant difference among groups. It is therefore unclear whether the number of IVIs is a risk factor for SE-IOP induced by anti-VEGF, and more RCTs are needed for confirmation. Wehrli^17^ found the rate of delayed OHT does not differ in eyes injected with mono-agent (bevacizumab or ranibizumab) and alternative agents. In the ranibizumab and bevacizumab subgroups, SE-IOP prevalence rates of 4.0% (8/196) and 9.5% (2/21) were obtained, respectively, with 7.2 ± 7.3 and 2.3 ± 1.5, respectively. Thus, more reliable data from RCTs should be provided to compare the different effects on SE-IOP of different anti-VEGF agents.

Theoretically, with increased follow-up duration and the accompanying increase of injection numbers, the cumulative effects of multiple anti-VEGF injections would appear. However, few studies have described such association. Therefore, we conducted a related meta-analysis, and found that RRs at 6, 12, and 23 months were 2.14 (CI 0.69–6.57), 3.15 (CI 0.99–10.09), and 3.48 (CI 1.38–8.78), respectively. In the included RCTs, the interval of injections was relatively fixed. Mitchell and Berger followed the schedule of 3 monthly +PRN, while others administered injections monthly or bimonthly. However, this meta-analysis could not determine injection-related cumulative effects on the increasing trend of SE-IOP, since different anti-VEGF agents were administrated in various subgroups. Aflibercept was used in the 6 and 23 month subgroups, and ranibizumab in the 12 month subgroup.

The high heterogeneity in prevalence assessment based on non-RCTs was the major limitation of this meta-analysis study, making the interpretation of outcomes complex. In non-RCT reports, different diagnostic criteria, baseline imbalance, IOP measuring methods, and various drug types and doses contributed to clinical heterogeneity. Since all the RCTs assessing anti-VEGF agents mainly aimed to evaluate the therapeutic and severe side effects, they were not strictly designed for controlling IOP. Well-designed RCTs with strict adherence to inclusion and exclusion criteria, standard IOP measurements, visual field assessments, and optic disk and nerve fibers thicknesses would provide more reliable evidence to support the current findings. We could not explain the different effects of ranibizumab, bevacizumab, aflibercept, and pegaptanib on SE-IOP. As this review focused on published data, publication bias resulting from unpublished data was inevitable although the funnel plot was asymmetric. For SE-IOP cases, detailed duration after injection was unclear. Whether IOP returned to baseline or not, and if not, when to intervene are areas deserving further exploration.

In conclusion, the included RCTs suggested a consistent 2-fold increase of SE-IOP risk after repeated intravitreal injection of anti-VEGF agents. SE-IOP beyond 21 mmHg and 5 mmHg above baseline occurred in up to 8.3% of patients, and patients with pre-existing glaucoma were more susceptible according to the current findings.

## Methods

### Search Strategy

Three databases (PubMed, EMBASE, and the Cochrane Library) were searched until January 28, 2016, using two domain terms: (1) anti-VEGF or equivalents (e.g., ranibizumab, bevacizumab, aflibercept, and pegaptanib); (2) intraocular pressure or equivalents (e.g., ocular tension). The results from each domain were combined with “AND”. Additionally, a manual search of relevant articles in reviews and original articles were conducted. All potentially related articles were retrieved and imported into EndNote X7, with duplicate studies manually removed.

### Inclusion and Exclusion Criteria

Published studies were included if they conformed to the following criteria: (1) parallel RCTs comparing IVI anti-VEGF with laser or sham injection, (2) non-RCTs focusing on SE-IOP after intravitreal injection of anti-VEGF with detail. The following exclusion criteria were adopted: (1) containing animal experiments; (2) reviews, comments, case reports, conferences, and unpublished or repeatedly published data; (3) anti-VEGF combined with intraocular surgery, definitely combined with systemically or ocular locally administered corticosteroids, and NVG; (4) transient rise in IOP for less than 1 day after IVI anti-VEGF. Two authors independently reviewed the titles and abstracts of the imported articles, and determined whether they were eligible. Full texts were read as necessary. Disagreements were discussed and resolved by consensus.

### Study Categorization and Risk of Bias Assessment

The included articles were categorized into RCTs and non-RCT studies according to study design.

The methodology quality of the selected RCTs was assessed based on the 7-point Jadad scoring system, which includes the following criteria: randomization, allocation concealment, double blinding, and description of withdrawals and dropouts. Scores were also based on the degree to which the following criteria were met: (1) description of the randomization method was appropriate; (2) description of the allocation concealment method was appropriate; (3) description of the double blinding method was appropriate. Matching each criterion counted as one point for study quality. A total score was then derived for each individual study to determine its quality.

### Data Extraction

Two authors independently screened, identified and extracted the search findings. The following data were extracted for each study: study design, publication year, origin, follow-up, disease, intraocular pressure and measuring method, injection number, criteria used to define an IOP increase, number of subjects with an IOP increase, and total number of subjects recruited. If patients in the RCTs crossed over from the control to active treatment group, we only included data collected before the crossover. Any discrepancies were resolved by consensus.

### Data Synthesis and Statistical Analysis

The data from all RCTs with balanced baseline measurements were included in the meta-analysis. RCTs were assessed using the Review Manager (RevMan) software, version 5.3 (Cochrane Collaboration, Oxford, UK). The data were combined using a fixed or randomized-effects model and dichotomous variable patterns depending on the significance of heterogeneity. In the meta-analysis of RCTs, the effect size of each study was presented as a risk ratio (RR) with 95% CI. The pooled effect size was considered significant when the 95% CI of the pooled risk ratio did not cross 1.0. Clinical heterogeneity was evaluated according to baseline and estimated statistical heterogeneity using the *I*^2^ statistic. Sensitivity analysis was also performed. Due to the limited number of RCTs, potential publication bias was not analyzed^43^.

The proportions of individuals with SE-IOP in retrospective case series and cohort studies were combined in this meta-analysis to provide a pooled SE-IOP prevalence. IOP prevalence increase was determined with STATA Version 14.0.367. Heterogeneity between studies was estimated using the *I*^2^ statistic. Sensitivity analysis was assessed by sequentially omitting one study. Potential publication bias was analyzed using the Egger’s test.

## Additional Information

**How to cite this article**: Zhou, Y. *et al*. Sustained elevation of intraocular pressure associated with intravitreal administration of anti-vascular endothelial growth factor: A Systematic Review and Meta-Analysis. *Sci. Rep.* **6**, 39301; doi: 10.1038/srep39301 (2016).

**Publisher's note:** Springer Nature remains neutral with regard to jurisdictional claims in published maps and institutional affiliations.

## Supplementary Material

Supplementary Information

## Figures and Tables

**Table 1:** Characteristics of the RCTs.

Study	Origin	Study Design	Baseline Characteristics				Disease	Arms of intervention			
			Follow-up (Month)	Excluded Glaucoma	Excluded steroids	Mean Age (SD)		Subjects	Treatment and Dose	Control	Jadad score
Mitchell^19^	USA	RCT	12	Yes	NO	62.9 ± 9.29	DME	115	IVR 0.5 mg 3 + PRN	laser + SI	7
VIVID 2011^21^	USA	RCT	23	NO	Yes	63.6 ± 8.3	DME	136 135	IVA 2 mgQ4 IVA 2 mgQ8	laser	3
VISTA 2011^20^	USA	RCT	23	NO	Yes	62.2 ± 9.7	DME	155 152	IVA2mgQ4 IVA 2 mgQ8	laser	3
Holz^22^	Germany	RCT	6	Yes	Yes	61.5 ± 12.9	CRVO	103	IVA 2 mg Q4	SI	4
Berger^23^	Canada	RCT	12	Yes	NO	61.7 ± 9.8	DME	75	IVR 0.5 mg 3 + PRN	laser	5

IVR, intravitreal Ranibizumab; IVA, intravitreal Aflibercept; 3 + PRN, three monthly loading dose followed by pro re nata treatment; SI, sham injection.

**Table 2:** Characteristics of non-RCTs.

Study	Origin	Study Design	Baseline Characteristics				Number with SE-IOP		SE-IOP Group	
			Disease	Mean Age (SD)	Sample size	Treatment and Dose (mg)	Total (%)	Glaucoma History	Mean Peak IOP (SD) mmHg	Mean Injection Number (SD)
Adelman^24^	USA	RS	AMD	N/A	116	IVR 0.5, IVB 1.5	4 (3.45%)	0	N/A	13.3 ± 4
Choi^25^	USA	RS	AMD	81 ± 10	155	IVB 1.25, IVB 0.5, IVP 1.6	9 (5.7%)	0	N/A	9.6 ± 7.7
Good^26^	USA	RS	AMD	N/A	215	IVB 1.25, IVR0.5	13 (6%)	7	29.2 ± 3.3	5 ± 1
Mathalone^14^	USA	RS	AMD	79 ± 8.3	201	IVB 1.25	22 (11%)	1	25.9 ± 3.3	5 ± 3.8^†^
Wehrli^17^	USA	RS	AMD	75.2	302	IVB, IVR	5 (1.6%)	2	N/A	8 ± 3^†^
Segal^28^	Israel	RS	AMD	N/A	528	IVB 1.25	19 (3.6%)	0	42.6 ± 10	7.8 ± 2.5
Hoang^27^	USA	RS	AMD	79.2 ± 12	449	IVB 1.25, IVR 0.5, IVP	32 (7.1%)	0	N/A	N/A
Kim^29^	Korea	RS	AMD RVO	67.2 ± 9.9	724	IVB 1.25, IVR 0.5	27 (3.7%)	8	19.6 ± 2.5	9.7 ± 5.9
Abdullah^30^	Saudi Arabia	RS	DME	61 ± 10.54	760	IVB 1.25, IVR 0.5	44 (5.8%)	0	N/A	3.6 ± 2.63
Nuzzi^31^	Italy	RS	AMD, DME, RVO, MMD	N/A	1173	IVB, IVR, IVP	40 (3.4%)	N/A	N/A	N/A
Baek^32^	Korea	RS	AMD DME	57.7 ± 27.8	152	IVB	9 (5.9%)	0	N/A	N/A
Agard^33^	France	PS	NA	77 ± 12.1	217	IVB 1.25, IVR 0.5	10 (4.6%)	4	29 ± 4	10.8 ± 11.6
Kiddee^34^	Thai-land	PS	AMD, DME, RVO	59 ± 12.8	70	IVB 1.25, IVR 1	4 (5.7%)	0	N/A	N/A
Bressler^35^	USA	PAFR	DME	63 ± 10	322	IVR 0.5				
Bakri^15^	USA	PAFR	AMD	N/A	749	IVR 0.3/0.5				
Boyer^16^	Brazil	PAFR	AMD	N/A	114	IVP 0.3				
Freund^36^	USA	PAFR	AMD	N/A	2411	IVA 2, IVR 0.5				

SE-IOP, sustained elevation of IOP; RS, retrospective study; PS, prospective study; PAFR, posthoc analysis from RCT; IVR, intravitreal Ranibizumab; IVA, intravitreal Aflibercept; IVP, intravitreal Pegaptanib; N/A, no available; ^†^Injection number used median (SD).

**Table 3:** Stratified analyses of sustained elevation IOP incidence.

Group	No. of Studies	Incidence of sustained elevation of IOP (95% CI)	P for heterogeneity	*I*^2^%
All	13	0.047 (0.037–0.058)	<0.001	67.5%
Diseases				
AMD	8	0.049 (0.032–0.065)	<0.001	76.4%
DME	1	0.058 (0.041–0.074)	/	/
AMD+DME+RVO	4	0.038 (0.028–0.047)	0.464	0.0%
No. of cases				
<300	7	0.058 (0.042–0.074)	0.232	25.8%
≥300	6	0.041 (0.037–0.058)	<0.001	77.4%
Diagnostic criteria				
>21 mmHg^24^	1	0.034 (0.001–0.068)	/	/
>25–26 mmHg^25^,^33^	2	0.050 (0.028–0.073)	0.611	0.0%
≥22 mmHg + above baseline ≥ 6 mmHg^14^,^26^	2	0.083 (0.035–0.131)	0.073	68.8%
≥22 mmHg + above baseline ≥ 6 mmHg OR > 26 mmHg^17^	1	0.017 (0.002–0.031)	/	/
>29 mmHg^28^	1	0.036 (0.020–0.052)	/	/
>25 mmHg + above baseline > 10 mmHg OR > 21 mmHg + above baseline > 5 mmHg^27^	1	0.071 (0.047–0.095)	/	/
above baseline ≥ 5 mmHg^18^,^31^,^32^,^34^	4	0.037 (0.029–0.045)	0.531	0.0%
above baseline > 6 mmHg or > 20% OR > 24 mmHg^30^	1	0.058 (0.041–0.074)	/	/
Sustained time				
4–6weeks^14^,^18^,^24^,^25^,^26^,^27^,^30^,^32^	8	0.058 (0.044–0.071)	0.038	52.8%
3–4weeks^33^,^34^	2	0.048 (0.024–0.073)	0.723	0.0%
4–6weeks or single visit^17^	1	0.017 (0.002–0.031)	/	/
single visit^28^	1	0.036 (0.020–0.052)	/	/
no mentioned^31^	1	0.034 (0.024–0.044)	/	/
