# Supplementary material for: Sustained Elevation of Intraocular Pressure Associated with Intravitreal Administration of Anti-vascular Endothelial Growth Factor: A Systematic Review and Meta-Analysis
Source: Sci Rep. 2016 Dec 21;6:39301. doi: 10.1038/srep39301 (PMC5175276; doi:10.1038/srep39301)

Sustained elevation of intraocular pressure associated with intravitreal administration of anti-vascular endothelial growth factor: A Systematic Review and Meta-Analysis

Yandan Zhou[1], Minwen Zhou MD[2], Shigang Xia[3], Qiancheng Jing[4], Ling Gao MD PhD[1]

[1]Department of Ophthalmology, the Second Xiangya Hospital, Central South University, Changsha, Hunan, China

[2]Department of Ophthalmology, Shanghai First People's Hospital, School of Medicine, Shanghai Jiao Tong University, Shanghai, China

[3]Department of Ophthalmology, the second hospital affiliated to University of South China, Hengyang, Hunan, China

[4] Department of Otorhinolaryngology, Xiangya Hospital,Central South University, Changsha, Hunan, China

Figure S1 Egger's tests indicated no evidence of publication bias in respect to the prevalence of SE-IOP in 13 non-RCTs

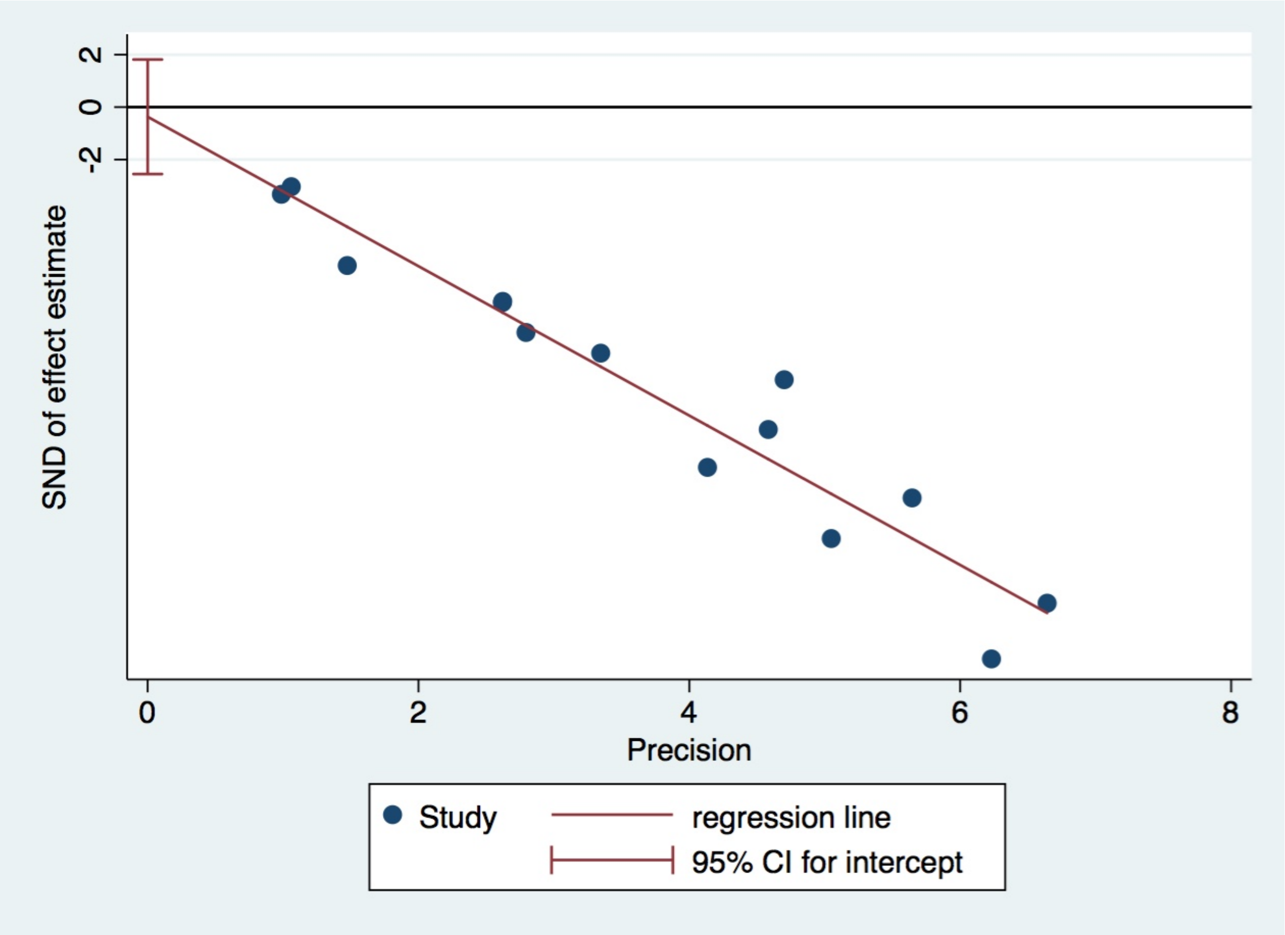

Figure S2 Pooled prevalence of SE-IOP induced by bevacizumab

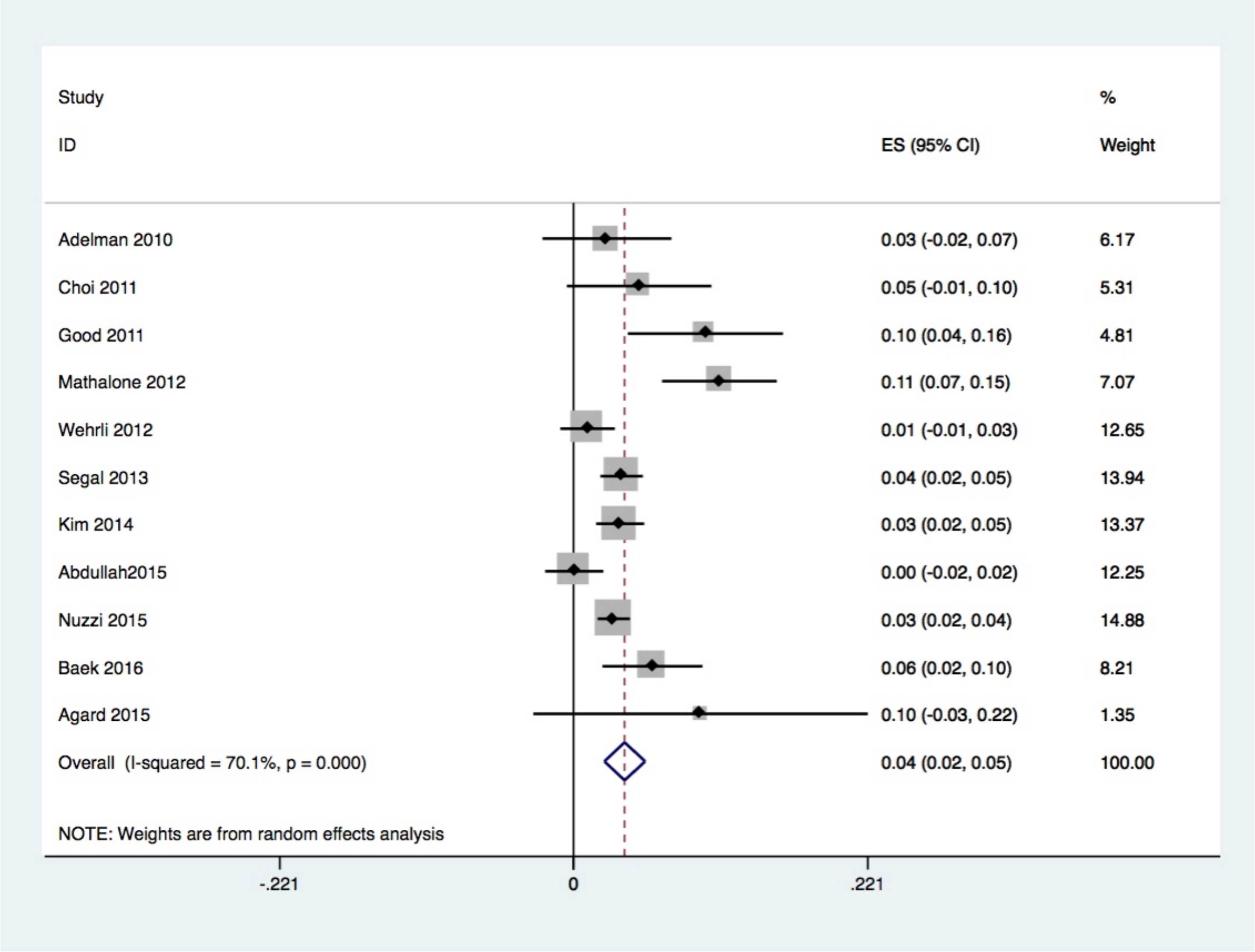

Figure S3 Pooled prevalence of SE-IOP induced by ranibizumab

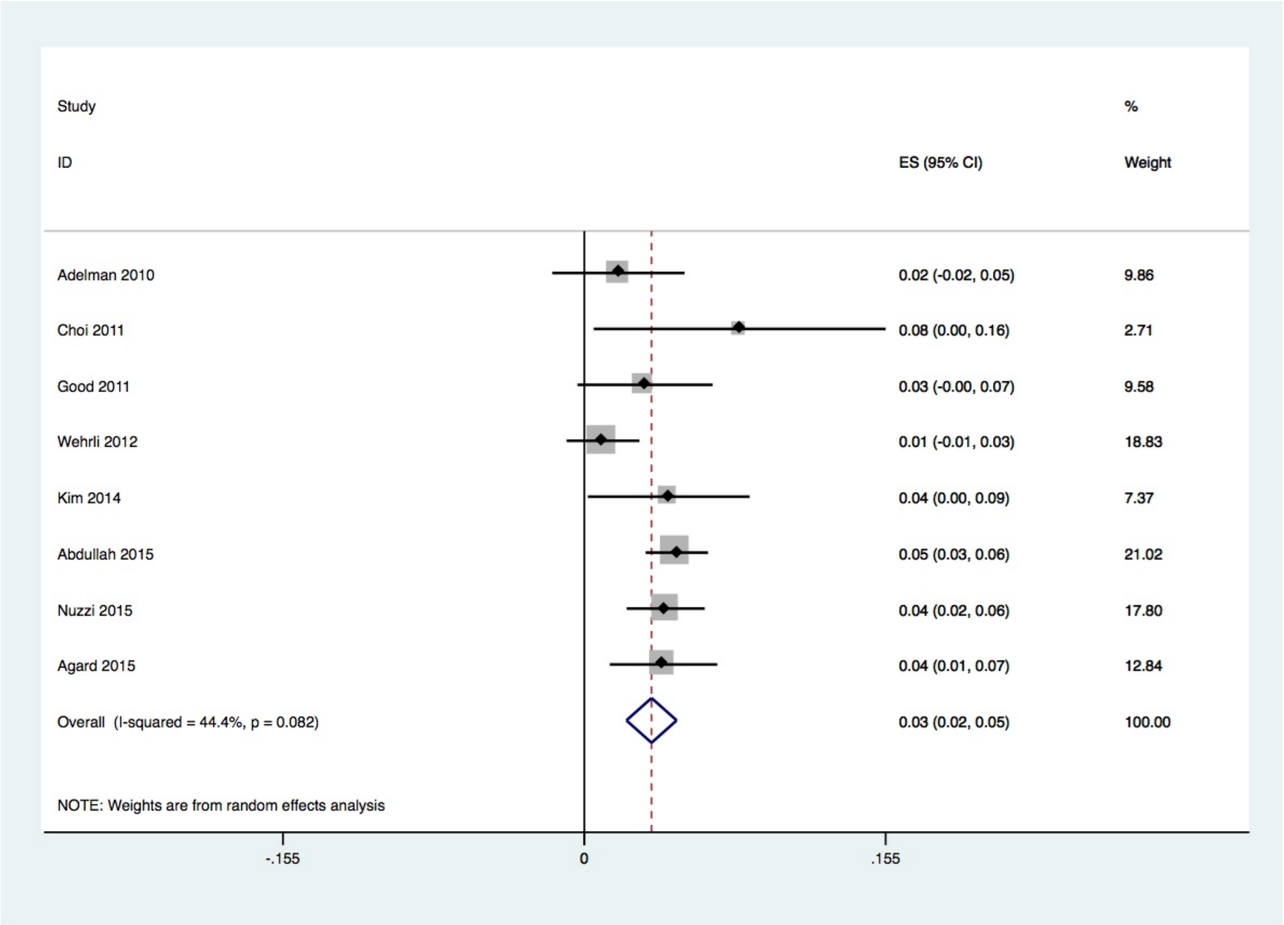

Supplement: Supplementary Information [file srep39301-s1.pdf]
